# Dietary Protein and Physical Exercise for the Treatment of Sarcopenia

**DOI:** 10.3390/clinpract14040117

**Published:** 2024-07-25

**Authors:** Rosarita Nasso, Antonio D’Errico, Maria Letizia Motti, Mariorosario Masullo, Rosaria Arcone

**Affiliations:** Department of Medical, Movement and Well-Being Sciences (DiSMMeB), University of Naples “Parthenope”, Via Medina 40, 80133 Napoli, Italy; rosaritanasso@gmail.com (R.N.); antonio.derrico002@studenti.uniparthenope.it (A.D.); letizia.motti@uniparthenope.it (M.L.M.); mario.masullo@uniparthenope.it (M.M.)

**Keywords:** sarcopenia, skeletal muscle, ageing, dietary proteins, physical exercise, myokines

## Abstract

Sarcopenia is a multifactorial age-related disorder that causes a decrease in muscle mass, strength, and function, leading to alteration of movement, risk of falls, and hospitalization. This article aims to review recent findings on the factors underlying sarcopenia and the strategies required to delay and counteract its symptoms. We focus on molecular factors linked to ageing, on the role of low-grade chronic and acute inflammatory conditions such as cancer, which contributes to the onset of sarcopenia, and on the clinical criteria for its diagnosis. The use of drugs against sarcopenia is still subject to debate, and the suggested approaches to restore muscle health are based on adequate dietary protein intake and physical exercise. We also highlight the difference in the amount and quality of amino acids within animal- and plant-based diets, as studies have often shown varying results regarding their effect on sarcopenia in elderly people. In addition, many studies have reported that non-pharmacological approaches, such as an optimization of dietary protein intake and training programs based on resistance exercise, can be effective in preventing and delaying sarcopenia. These approaches not only improve the maintenance of skeletal muscle function, but also reduce health care costs and improve life expectancy and quality in elderly people.

## 1. Introduction

In recent years, in many countries, the improvement of social-economic conditions and advances in biomedical sciences have led to a longer lifespan in humans [1,2]. In these societies, elderly people, older than 60–65 years, represent a growing proportion of the population; this entails considerable health care costs [3,4].

Ageing is often accompanied by the onset of pathologies and metabolic disorders, which, above all, influence the movement and cognitive functions of the elderly [5,6,7,8,9,10,11]. Skeletal muscle tissue represents approximately 40–50% of the entire body mass and, therefore, the most abundant tissue and performs a fundamental function [12,13]. Skeletal muscle is not only responsible for locomotion; it also represents a reserve source of amino acids for protein metabolism. It is also involved in metabolic regulation with other organs and tissues [12,14,15] acting as an endocrine organ through exercise-induced myokine secretion [16,17]. In elderly people, sarcopenia is a common skeletal muscle disorder that is characterized by a decrease of muscle mass and functions [7,18,19], causing an impairment of mobility, risk of falls, hospitalization, and mortality [20,21].

The clinical needs and demand for the care of elderly people suffering from sarcopenia have a major impact on health care costs [3,4]. In the last decades, considerable effort has been devoted to the identification of factors and physio-pathological mechanisms involved in sarcopenia to develop strategies for its prevention, delay, and treatment. Sarcopenia is a multifactorial disorder that it is also linked to other diseases with a low grade of chronic inflammation such as diabetes, inflammation, obesity, and cancer [10,19,22]. Its diagnosis, as well as the efficacy of therapeutic interventions, still remain elusive [23,24,25,26].

The most promising approaches for the delay and management of sarcopenia are based on adequate diet and physical exercise [27,28,29,30], as these also positively affect mental health and cognitive functions [9,31]. However, the potential preventive and beneficial effects of diet alone or in combination with exercise training still require further investigation. 

In this article, we therefore aim to highlight recent scientific evidence on the pathophysiology of sarcopenia in ageing, on diagnostic techniques, and on the molecular biomarkers linked to muscle health contributing to its diagnosis. We then focus on the effect of the amount and quality of protein intake, in particular comparing the food source (plant derived vs. animal). Finally, we evaluate the effect of physical exercise on counteracting sarcopenia by focusing on the role of exercise-induced myokines, which are key mediators of the beneficial effect induced by physical exercise on muscle health. 

## 2. Pathophysiology of Sarcopenia

**Figure 1:**
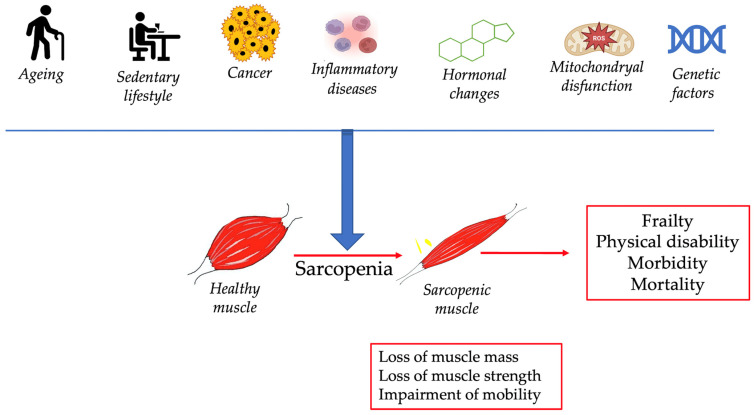
Schematic representation of major factors contributing to sarcopenia and effect on human health.

In humans, ageing determines the gradual and slow alteration of cellular and metabolic processes that affect various organs and tissues [10,18,32]. Age-related impairments encompass a wide range of factors, including hormonal changes, inflammation, cancer, genetic modification, lifestyle, and environmental exposure [10,18,22,33,34]. All of these factors contribute to a decline and slowdown of physiological functions, an increased risk of diseases, and an impairment of cognitive abilities [9,11,13,35,36,37] (Figure 1). 

The term, sarcopenia, refers to a progressive and generalized skeletal muscle disorder that is characterized by the loss of muscle mass, strength, and functions [7,18,38]. Although sarcopenia is mainly considered a geriatric syndrome, it has been classified as a primary or age-related disease in the elderly, or a secondary pathology that occurs in young people with other diseases such as diabetes, obesity, and cancer [7,10,22,39,40,41]. Sarcopenia is linked to an increased risk of human mobility problems and difficulty in walking, leading to falls and hospitalization that greatly affect quality of life, especially for the elderly [21].

### 2.1. Sarcopenia in Ageing

Sarcopenia is a multifactorial disease [10,13,18,22], and although its underlying pathophysiological mechanisms still need further investigation, several factors, mostly related to ageing, have progressively emerged.

The major factors include the following:

− Hormonal changes with a decline in different anabolic hormone levels such as growth hormone (GH), sex hormones (testosterone, estrogen), insulin-like growth factor-1 (IGF-1), and dehydroepiandrosterone (DHEA); this decline impacts muscle protein synthesis. Among these, testosterone and GH are powerful anabolic hormones for their ability to promote protein biosynthesis and subsequent muscle mass development [8];
− Mitochondrial dysfunction and biogenesis disorders cause a decrease in ATP production and an increase in ROS levels that is linked to cell senescence; these alterations also impair skeletal muscle contraction since most of ATP synthesis occurs by means of oxidative mechanisms in the mitochondrion, through oxidative phosphorylation [42,43];
− Chronic and low-grade systemic inflammation termed “inflammaging” is also associated with various age-related diseases [33] characterized by an increased level of inflammatory cytokines such as the tumor necrosis factor-α (TNF-α), Interleukin-6 (IL-6), and Interleukin-1α (IL-1α) [34,44].

Finally, sarcopenia has also been linked to cognitive decline and dementia found in elderly people [9,45,46]. In fact, several studies have investigated the relationship between the decrease in skeletal muscle mass and the increased risk of dementia in elderly people, reporting the association between reduction of muscle mass and cognitive decline in older people (≥60 years) [9,11,46,47]. The molecular mechanism underlying this association involves inflammatory factors and myokines such as C-reactive protein (CRP) and IL-6, which are both linked to the reduction of skeletal mass [48,49,50,51].

### 2.2. Sarcopenia in Cancer

Among inflammatory diseases, sarcopenia is very common in cancer patients since severe malnutrition, i.e., cachexia with a concomitant reduction of skeletal muscle mass, affects overall health and treatment efficacy in many of them [52,53,54,55]. Cancer cachexia also induces systemic inflammation and metabolic alterations, and, in these patients, severe sarcopenia is caused by poor food intake due to loss of appetite and the side effects of therapy [55,56,57,58]. Sarcopenia also causes depression in cancer patients, adverse clinical outcomes, and increased toxicity due to chemotherapy drugs, which can even result in the impossibility of continuing anti-tumor therapies [59]. Cancer patients with sarcopenia are prone to numerous complications such as infections and perioperative problems, which further compromise their clinical condition [60] because sarcopenia causes a progression of the disease that, in turn, induces physical inactivity and loss of appetite.

In patients suffering from various forms of tumors, those with sarcopenia usually show reduced survival, as demonstrated by both non-metastatic patients undergoing therapy and metastatic ones [61]. Today, in the clinical management of cancer patients, DNA screening is being performed to identify target genes involved in effective therapy; this genetic approach also allows for the identification of new target genes that can be used to develop specific novel inhibitors [62,63]. In addition, elderly patients need to be subjected to less aggressive therapies as they present various comorbidities, including sarcopenia. Indeed, sarcopenia is considered a prognostic factor in the survival of elderly patients with numerous pathological conditions, including cancer [6,60,64,65,66]. However, in some cancer patients, sarcopenia is difficult to detect, especially when physical inactivity and malnutrition coexist, causing either a decrease in muscle growth or an increase in adipose tissue, leading to sarcopenic obesity that is associated with a poor prognosis compared to that of non-sarcopenic obese patients [54,67,68].

Finally, since sarcopenia has prognostic significance in cancer patients, its early identification may allow for appropriate intervention.

Some cancer patients have been subjected to physical exercise training protocols based on progressive resistance exercises to strengthen large muscle groups [69]. This type of activity exerts either a decrease in disease-related fatigue and anxiety or an increase in weight and body mass index (BMI), leading to an improvement in muscle function [70,71].

However, in cancer patients, the loss of muscle mass is only the main symptom of neoplastic cachexia, as this disease is linked to inflammation, anorexia, and muscle proteolysis that affect the whole body [59,61,72,73]. In patients with cachexia, the distinctive element is the atrophy of the skeletal muscle in the presence or absence of fat loss [68]. Cachexia is very common, especially in patients suffering from tumors of the gastrointestinal tract and lungs [39,74]. There are three different stages in cachexia: pre-cachexia, cachexia, and refractory cachexia [54]. The specific stage can be assessed by analyzing the availability of the body’s energy and protein reserves and simultaneously assessing the severity of weight loss. Therefore, the clinical management of the patient should always take reduced food intake, the activation of catabolic metabolism, and the evaluation of muscle mass, strength, and function into consideration [54,72]. It is, however, important to highlight that tumor cachexia is not always related to weight loss, and, in this case, it is called “hidden cachexia” [75,76]. Understanding the health status of cancer patients is fundamental for evaluating their nutritional needs together with an appropriate physical activity intervention, which can also exert beneficial effects on inflammatory status [52,53,77].

### 2.3. Clinical Diagnosis of Sarcopenia

Since sarcopenia is characterized by the decrease of skeletal mass and strength that impairs physical performance, its clinical diagnosis is based on techniques that allow for the assessment of the parameters related to all these alterations.

Currently, imaging techniques and clinical evaluation are the main approaches employed [19,78]. They include the following:

− Dual-energy X-ray absorptiometry (DXA) scans, bioelectrical impedance analysis (BIA), and MRI/CT scans for the determination of lean skeletal muscle mass;
− Grip strength tests, knee flexion/extension strength, and walking speed for the assessment of muscle strength, with specific cut-off points indicating reduced muscle strength;
− The Short Physical Performance Battery (SPPB), gait speed, and Timed Up and Go (TUG) tests for the evaluation of functional physical performance; low scores on these tests may indicate impaired physical performance.

Although the overall assessment of these measurements contributes to the diagnosis of sarcopenia, there is currently no consensus definition of sarcopenia or of the clinical parameters that contribute to its diagnosis [5,19,78,79].

In addition, clinically, sarcopenia differs from other conditions such as dynapenia, which indicates age-related muscle weakness, but it is not associated with neuromuscular disorders; dynapenia is usually revealed by a dynamometry test, and it can, therefore, be used as an initial screening mechanism for sarcopenia detection [80,81].

A number of guidelines for the diagnosis of sarcopenia correlated by tests and criteria have been proposed by different organizations. Among these, in 2019, consensus guidelines were provided by the European Working Group on Sarcopenia in Older People (EWGSOP) [79]. Table 1 summarizes the diagnostic criteria that are habitually employed for the clinical diagnosis of sarcopenia.

### 2.4. Molecular Biomarkers and Genetic Factors of Sarcopenia

In addition to clinical diagnosis, many studies have described molecular biomarkers that enable the early diagnosis of sarcopenia and allow for an appropriate therapeutic approach or intervention [82,83,84]. 

Since sarcopenia is a multifactorial disease, biomarkers mainly correlated to various cellular metabolic processes and muscle health play a crucial role; for instance, those involved in inflammation are mainly C-Reactive Protein (CRP), IL-6, and TNF-α, and others linked to muscle integrity (Creatine Kinase, CK) or to oxidative stress (8-isoprostens) [82]. 

Biomarkers linked to muscle health involve amino-terminal pro-peptide of type-III procollagen, c-terminal agrin fragment-22, osteonectin, irisin, fatty acid-binding protein-3, and macrophage migration inhibitory factor [84].

On the other hand, genetic factors reported as sarcopenia biomarkers have also been identified such as angiotensin I-converting enzyme I (ACE), myostatin (MSTN), alpha actinin 3 (ACTN3), ciliary neurotrophic factor (CNTF), vitamin D receptor (VDR), insulin-like growth factor 1 (IGF1), and IL-6 [85].

## 3. Current Strategies for the Prevention and Treatment of Sarcopenia

Numerous efforts have been devoted to identifying pharmacological therapies for the prevention and treatment of sarcopenia through the use of anabolic steroids, selective androgen receptor modulators, and inhibitors of myostatin or its bio-signaling pathway [24,25,26].

Many pharmacological interventions have been conducted by means of clinical trials in sarcopenic older adults to evaluate the efficacy of drugs on muscle mass, strength, and physical performance. Among the pharmaceutical treatments, myostatin inhibitors, anabolic or androgenic steroids, growth hormones, angiotensin-converting enzyme (ACE) inhibitors, troponin activators, appetite stimulants, activating II receptor drugs, and β-receptor blockers have not been fully effective, also leading to side effects [25,86]. However, strategies based on nutritional intake and physical exercise have demonstrated greater benefit in the improvement of lean body mass and muscle strength than those obtained via pharmacological treatments [27,29,87]. In addition, up to now, no drugs have been approved for sarcopenia treatment [88]; conversely, the supplementations of protein, vitamin D, and resistance exercise have emerged as effective strategies [23,89].

### 3.1. Dietary Protein Strategy against Sarcopenia

In ageing, the loss of muscle mass and function is linked to alterations of protein metabolism caused by an imbalance between protein synthesis and breakdown, which greatly influences the plasticity and function of this tissue and its crosstalk with other organs such as the liver, adipose tissue, and bones [12,14,90,91,92]. This muscle impairment, termed primary sarcopenia, is mostly due to the decrease in anabolic hormones and causes less responsive skeletal muscle compared to that of younger adults [7,18,93]. In this metabolic condition, dietary protein intake plays a crucial role in maintaining muscle health, and efforts have been devoted to investigating whether there are metabolic or nutritional differences between animal and plant diet protein [87,94,95,96,97]. In addition, to date, the spread of vegetarian and vegan diets for ethical and environmental reasons makes it important to define the nutritional and metabolic aspects linked to sarcopenia.

#### Nutritional Classification of Proteinogenic Amino Acids in Humans

In humans, proteins are made up of 21 different amino acids (AAs), including the 21st AA selenocysteine (Table 2, which also act as the nitrogenous backbones for hormones and neurotransmitters [98]. Nine of these AAs have been classified as essential or indispensable (EAAs) (Histidine, Isoleucine, Leucine, Lysine, Methionine, Phenylalanine, Threonine, Tryptophan, and Valine) because they cannot be synthesized endogenously and, therefore, must all be introduced through diet protein [99,100]. Three EAAs (Isoleucine, Leucine, and Valine) represent Branched Chain Amino Acids (BCAAs), which play key roles in protein metabolism and as energy substrates in the skeletal muscle [101,102]. Six AAs are defined as conditionally essential (Arginine, Cysteine, Glutamine, Glycine, Proline, and Tyrosine) because their synthesis can only occur under pathophysiological conditions (severe catabolic condition) or they cannot be produced in adequate amounts [103]. The six non-essential AAs (Alanine, Aspartic acid, Asparagine, Glutamic acid, Serine, and Selenocysteine) are synthetized by intermediates of metabolic pathways such as the citric acid cycle and AA transamination reactions [99,100].

Muscle health and physical performance depend above all on AA intake, either of their quantity or quality. The daily protein requirement varies mainly depending on age, sex, physical activity, and health state. Young people and adults need a protein intake of approximatively 0.7 g/kg body weight/day; in older people, the European Society for Clinical Nutrition and Metabolism (ESPEN) recommends a diet containing at least 1.0–1.2 g protein/kg body weight/day for healthy older adults (≥60 years old), whereas a higher protein intake (1.2–1.5 protein/kg body weight/day) may be beneficial for elderly and sarcopenic adults with acute or chronic diseases, including cancer [93,96,104,105].

Currently, the question as to whether animal or plant protein sources may differentially prevent and/or influence muscle health and sarcopenia is still under investigation. Amino acids are provided by both animal- and plant-based diets; however, as concerns the content of EEAs (Table 3), their amount and quality vary depending on the specific animal or plant food.

Leucine plays a significant role in muscle protein synthesis, but some plant-based protein sources show lower Leucine content compared to that of animal-based sources [106,107]. Including Leucine-rich foods such as peanuts, soybeans, lentils, and chickpeas in the diet can help address this concern.

Animal proteins are considered high-quality, or complete, proteins because they contain all nine essential amino acids; vice versa, vegetable proteins are considered low-quality, or incomplete, because they lack one or more essential amino acids. Although plant-based food shows an incomplete amino acid profile due to the lack of some EAAs found in animal sources and lower digestibility, growing evidence indicates that plant proteins may be useful in age-related diseases in older adults; in particular, improving body composition and physical function [108]. Animal- and plant-based diets not only differ in the quality and amount of the AAs, but also in regard to digestibility, adsorption kinetics, and interactions between nutrients and the food matrix [97].

It is not only the protein content, but also the various components of a plant-based diet rich in fruits and vegetables that can prevent and mitigate sarcopenia by providing additional nutrients such as vitamins, minerals, and phytochemicals. Among the phytochemicals, polyphenols show antioxidant and chemo-preventive properties [95,109,110], improving muscle health by reducing the generation and inflammation of reactive oxygen species (ROS) [94,97]. These antioxidant and anti-inflammatory properties may help to reduce chronic inflammation associated with sarcopenia, supporting better muscle function.

A further consideration of the differences between animal and plant AA sources concerns the digestibility of the proteins containing them. Plant proteins contain other components that can reduce complete AA absorptivity; in fact, protein digestibility also depends on the cooking phase, as well as on other factors such as soaking, fermenting, and sprouting, that can impair the process [95].

Since nutrition interventions are the major strategic approaches to preventing, delaying, and counteracting sarcopenia, various randomized clinical trials have been conducted to evaluate the effect on muscle mass, strength, and function [86,87,111]. Table 4 summarizes dietary interventions, and their effects reported on sarcopenia.

### 3.2. Physical Exercise against Sarcopenia 

Among the factors involved in sarcopenia, lifestyle plays a major role. In fact, a sedentary lifestyle is responsible for muscle weakness, and it is related to a reduction of muscle mass and strength. Vice versa, a dynamic lifestyle can prevent decay in muscle mass and strength; it would, therefore, appear that exercise training is useful for both younger and older people [36].

At the molecular level, intense and prolonged physical exercise promotes muscle protein synthesis and increases the catabolism of fatty acids for ATP production, leading to a reduction of adipose tissue [112,113]. In addition, physical exercise combined with adequate protein intake amplifies protein synthesis and inhibits protein catabolism due to high blood insulin concentration [114].

The health benefits of regular exercise are not limited to skeletal muscle, but involve the whole body, ameliorating some chronic pathologies such as cardiovascular diseases, hypertension, hyperlipidemia, metabolic syndrome, cancers, and diabetes. In older people, physical activity can prevent and contrast age-related sarcopenia and loss in muscle mass; it can also help to preserve or increase bulk and muscle strength [40,115]. Several types of exercise training have been described for the prevention and treatment of sarcopenia [114], such as resistance, endurance, aerobic, balance, flexibility, functional, and whole-body vibration training (WBVT). These activities are summarized in Table 5.

During the training program, the intensity, duration, and resistance can be gradually increased to enhance the effect on skeletal muscle.

However, a specific exercise training program must be planned by a fitness expert able to consider the health state of the sarcopenic subject, age, sex, and other clinical conditions, such as the presence of other pathologies (i.e., cancer, obesity, diabetes, neurodegenerative disorders) or specific disabilities.

### 3.3. Exercise-Induced Myokines and Sarcopenia

In recent years, skeletal muscle has also been defined as an endocrine organ for its ability to secrete exercise-induced proteins and peptides named myokines, acting as key mediators of the muscle and whole-body health [17,125]. Myokines are also involved in sarcopenia, acting in an autocrine, paracrine, and endocrine manner and playing a role in the regulation of muscle mass, energy metabolism of glucose, fatty acids, proteins, and inflammation, allowing a crosstalk between skeletal muscle and other organs such as the liver, brain, and adipose tissue [15,126]. Many myokines are pleiotropic factors, and some of them, like Interlukin-6 (IL-6), insulin-like growth factor-1 (IGF-1), IL-15, irisin, fibroblast growth factor (FGF)-21, brain-derived neurotrophic factor (BDNF), myostatin, and IL-10, are involved in muscle cell proliferation, differentiation, mitochondrial function, inflammation, and metabolic homeostasis.

Myokine signaling pathways are also involved in the proliferation and differentiation of muscle cells, muscle atrophy, increased mitochondrial function, decreased inflammation, and metabolic homeostasis [15,126,127], and they can exert either pro- or anti-inflammatory signaling roles [128].

IL-6 regulates muscle and whole-body glucose and lipid metabolism, and it can also have both pro-inflammatory and anti-inflammatory effects depending on the cytokine release mode, target cells, and simultaneous presence of other cytokines and the inflammatory C-reactive protein (CRP) associated with an increased risk of muscle strength loss [15,22].

IGF-1 exerts a major role in muscle growth, hypertrophy, regeneration, and differentiation through the induction of satellite cell proliferation and differentiation [15,22]. IGF-1 counteracts the age-dependent reduction of GH/IGF-1 axis, causing a decrease in protein anabolism in the skeletal muscle of sarcopenic patients [8,15].

IL-15 increases muscle growth and participates in the crosstalk between skeletal muscle and adipose tissue, reducing adipose tissue mass; the decreased plasma levels of IL-15 have been associated with sarcopenia and obesity, thus suggesting that IL-15 may be a promising candidate for the treatment of sarcopenia [22,42,127].

FGF-21 acts as a regulator of muscle growth, inflammation, metabolism, and premature ageing, and a positive correlation has been found between serum FGF-21 levels and both sarcopenia and sarcopenic obesity [20,22,127].

Irisin regulates myogenic differentiation, mitochondrial function, and metabolic homeostasis. The exercise-induced expression of irisin, or intervention with exogenous irisin, can delay the progression of this chronic disease; conversely, its suppression or knockout leads to several chronic diseases, including sarcopenia [22,42,129].

BDNF, a neurotrophic factor produced both in the brain and in skeletal muscle, regulates neuronal function, and it is also implicated in the regulation of energy homeostasis and body weight, affecting myogenesis and activating satellite cells in skeletal muscle [22]. In addition, the BDNF signaling pathway plays an essential role in the regulation of neuromuscular function during ageing, which may have implications for the onset of sarcopenia and sarcopenic obesity [130].

Myostatin, known as growth differentiation factor 8 (GDF-8), is a member of the transforming growth factor (TGF-α) superfamily, and it is expressed primarily in skeletal muscle, where it acts as a negative regulator of muscle mass growth and development [22]. Serum levels of myostatin increase with age, and they are inversely correlated with skeletal muscle mass, leading to sarcopenia [15]. In addition, myostatin may inhibit the biosynthesis of irisin, contributing to an increase in fat mass and a decrease in muscle mass, predisposing older people to sarcopenic obesity [22]. Therefore, the inhibition of myostatin represents a plausible option for the treatment of sarcopenia [15].

IL-10 reveals an anti-inflammatory function by suppressing macrophage activation, and evidence points to an increase in both older mice and elderly people [127]. Growth differentiation factor 15 (GDF15) is involved in the regulation of metabolic health and energy metabolism, and its levels are elevated in diseases associated with muscle weakness, such as sarcopenia and mitochondrial myopathy [130,131].

The reduction of muscle mass in sarcopenia and the effects of a sedentary lifestyle impair the circulating level of myokines, as demonstrated by the decrease of IGF-1, IL-15, irisin, FGF-21, and BDNF, and the increase of myostatin, IL-10, and TNF-α [22,130,132,133]. However, this alteration of myokine levels can be attenuated by regular physical activity training [128,132]. Taken together, evidence indicates that myokines may act either as potential diagnostic biomarkers or as therapeutic targets in sarcopenia and its related diseases such as obesity and cancer [133].

## 4. Discussion and Conclusions 

An understanding of the cellular and molecular mechanisms underlying sarcopenia is crucial for its prevention and mitigation in elderly individuals. Evidence has demonstrated that sarcopenia is a multifactorial disease since numerous factors, such as hormonal changes, inadequate nutrition, and physical inactivity, greatly contribute to its development. However, among these, impaired muscle protein metabolism and a sedentary lifestyle with reduced physical activity greatly contribute to the development of sarcopenia.

Early diagnosis of sarcopenia is important for preventing and delaying the disease in the elderly, and especially in patients with chronic diseases closely related to an inflammatory state such as cancer, diabetes, obesity, and metabolic syndrome. Currently, the diagnosis of sarcopenia is established through the overall assessment of physical performance tests and diagnostic investigations that are interpreted by applying key diagnostic criteria. However, it is difficult to establish a precise diagnosis in the presence of other pathologies causing muscle weakness and impaired physical mobility. For this reason, great efforts have been undertaken to identify biomolecular markers closely related to skeletal muscle integrity and metabolism that could contribute greatly to an early diagnosis.

To date, the identification of specific molecular biomarkers of sarcopenia is still under investigation, aiming at better defining their specificity and threshold values, which depend on various factors, including ethnicity, age, gender, diet, and lifestyle.

Currently, approved pharmacological therapies that are both efficient and safe when dealing with sarcopenia are not available yet.

**Figure 2:**
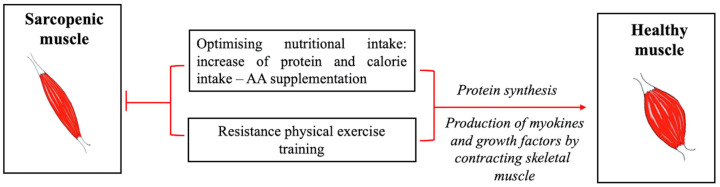
Schematic representation of strategies based on optimizing nutritional intake and resistance physical exercise for preventing, delaying, and counteracting sarcopenia in humans.

In fact, as with many other multifactorial diseases, the pharmacological approach to sarcopenia has not yielded encouraging results, and there is, therefore, still a need to identify drugs that are both effective and safe. However, key strategies based on lifestyle modification, including a balanced diet and regular exercise, have been proven effective for the prevention and mitigation of sarcopenia in older individuals (Figure 2).

Regarding the nutritional sources of protein, the contribution of diets containing reduced, or no animal foods needs to be better understood with regards to ageing and its associated diseases. Maintaining healthy muscles through proper nutrition and exercise is crucial, especially for cancer patients dealing with sarcopenia. Resistance training reduces muscle atrophy by promoting muscle protein synthesis during ageing. Therefore, a diet with both adequate food protein and physical exercise is crucial for maintaining a healthily muscled body system and preventing, delaying, and counteracting sarcopenia, especially in elderly people.

Further research in these fields will provide new insight into the prevention and management of sarcopenia.

## Figures and Tables

**Table 1:** Criteria for the diagnosis of sarcopenia provided by EWGSOP: parameters, tests/techniques, and cut-off values, as previously reported [79].

Parameter	Test—Technique	EWGSOP 2019 Cut-off Value
Muscle Strength	Hand Grip Strength (by hand dynamometer)	<27 kg (men) <16 kg (women)
Muscle Quantity/Quality	DXA (Appendicular Lean Mass, measures appendicular lean mass adjusted for height)	<7.0 kg/m^2^ (men) <5.5 kg/m^2^ (women)
	BIA (Skeletal Muscle Mass Index, assesses skeletal muscle mass)	<7.0 kg/m^2^ (men) <5.7 kg/m^2^ (women)
Physical Performance	Gait Speed (Measured over 4 m walk)	≤0.8 m/s
	Short Physical Performance Battery (SPPB)	≤8 points
	Timed Up and Go (TUG): time to stand from a seated position, walk 3 m, turn, walk back, and sit down	≥20 s
	Chair Stand Test: time to rise from a chair 5 times consecutively	>15 s (5 rises)

**Table 2:** Nutritional classification of proteinogenic amino acids in Essential (EAA) and Branched Chain Amino Acids (BCAAs), conditionally essential and non-essential in humans, as previously reported [98].

Essential AA	Conditionally Essential AA	Non-Essential AA
Histidine	Arginine **	Alanine
Isoleucine *	Cysteine	Aspartic acid
Leucine *	Glutamine	Asparagine
Lysine	Glycine	Glutamic acid
Methionine	Proline	Serine
Phenylalanine	Tyrosine	Selenocysteine
Threonine		
Tryptophan		
Valine *		

* Indicates Branched Chain Amino Acid (BCAA). ** Indicates essential AA in childhood and some pathological conditions.

**Table 3:** EAAs in plant and animal food sources in common human diets, as previously reported [99].

EAA	Plant Food Source	Animal Food Source	Daily Intake (mg/kg Body Weight) **
Histidine	Lentils, quinoa, chickpeas, hemp seeds	Beef, chicken, fish	10
Isoleucine *	Soybeans, lentils, cashew nuts, oats	Eggs	20
Leucine *	Peanuts, soybeans, lentils, chickpeas	Beef, chicken, fish,	39
Lysine	Quinoa, lentils, black beans, pumpkin seeds	Red meat, poultry, dairy produce	30
Methionine	Brazil nuts, sunflower seeds, oats, spirulina	Eggs, fish	15 (including cysteine)
Phenylalanine	Pumpkin seeds, soy products, almonds, chickpeas	Eggs, milk, cheese	25 (including tyrosine)
Threonine	Soybeans, lentils, sesame seeds, quinoa	Lean meats, cottage cheese	15
Tryptophan	Pumpkin seeds, chia seeds, soybeans, sunflower seeds	Turkey, chicken, milk	4
Valine *	Peanuts, lentils, soy-beans, mushrooms	Meat, dairy produce	26

* Indicates Branched Chain Amino Acid (BCAA). ** Indicates recommended daily intake for healthy adults by the World Health Organization (WHO).

**Table 4:** Dietary interventions, nutrient components, and properties/effects on sarcopenia.

Dietary Intervention	Nutrient Components	Properties and/or Effects	References
Increase of dietary protein intake and AAs supplementation	EAAs, BCAAs	Increase of muscle protein biosynthesis; improvement of muscle mass and function	[67,90,93,96]
Food supplements	Leucine; β-hydroxy-β-methylbutyrate (HMB)	Increase of muscle protein biosynthesis and reduction of muscle protein breakdown	[106,111]
	Vitamins (C, D, E)	Antioxidant properties; decrease of inflammation and muscle damage	[95,99]
Naturally derived food supplements	Plant polyphenols	Antioxidant, anti-inflammatory properties; protection against muscle damage	[94,97,99]

**Table 5:** Outline of types, volume, and benefits of physical exercises that are recommended for the prevention and treatment of sarcopenia.

Type of Physical Exercise	Description	Volume and Frequency	Benefits	References
Resistance training	Exercises with weights, resistance bands, or body weight	2–3 times per week, 1–3 sets per exercise, 8–12 repetitions per set	Increases of muscle mass, strength, bone density, reducing the risk of osteoporosis-related fractures	[28]
Endurance training	Chest press exercises, dorsal and rowing machines for upper body, leg press, leg extension and knee flexibility for lower body	2–3 times per week, 20–60 min per session, with 1–2 sets and 8–10 repetitions to 2–3 sets and 6–8 repetitions	Improves cardiovascular health, aids in weight management, and boosts endurance	[116,117]
Aerobic training	Stationary cycling and walking on treadmill	2–3 times per week 20–45 min	Improves cardiovascular health, muscle mass and strength, aerobic capacity	[118,119]
Balance training	Activities for stability improvement, such as tai chi, yoga, or specific balance exercises	2–3 times per week, 20–30 min per session	Enhances coordination, stability and proprioception, reducing the risk of falls and injuries	[119,120]
Flexibly training	Stretching exercises to enhance range of motion and flexibility	2–3 times per week, 10–15 min per session, holding each stretch for 15–30 s	Improves flexibility of joints and reduces muscle stiffness	[121]
Functional training	Exercises mimicking daily activities, often integrating strength, balance, and coordination	2–3 times per week, 20–30 min per session	Enhances ability to perform daily tasks, improves overall function	[122]
Whole body vibration training (WBVT)	Participants squat or stand on the vibrating platforms	Vibration frequency and amplitude can be set differently depending on the machine for a safe effect on skeletal muscles (12–300 Hz) Time duration: variable (12–15 min)	Enhances muscle strength, balance, bone density, flexibility, reducing the risk of falls and injuries	[123,124]

## Data Availability

No new data were created or analyzed in this study. Data sharing is not applicable to this article.
